# 603 Disparities in Pavement Burn Severity and Outcomes: A Comparative Analysis of Homeless and Non-Homeless Patients

**DOI:** 10.1093/jbcr/iraf019.232

**Published:** 2025-04-01

**Authors:** Henry Krasner, Emma Chevalier, David Slattery, Syed Saquib

**Affiliations:** Burn Center at University Medical Center; University of Nevada, Las Vegas; Kirk Kerkorian School of Medicine at UNLV; University of California Irvine Medical Center

## Abstract

**Introduction:**

In some regions, extreme heat can result in pavement temperatures high enough to cause severe burn injuries upon skin contact. This risk is elevated for persons experiencing homelessness (PEH) who may lack adequate clothing and shelter, increasing exposure to hot pavement, and have other risk factors including substance use and loss of consciousness. While prior studies have shown worse outcomes for PEH due to delays in care and higher susceptibility, there is a lack of data on the impact of pavement burns specifically within this population. This study aims to explore burn severity and hospital outcomes in homeless versus non-homeless (NH) individuals with pavement burns. We hypothesized that PEH would have larger burn size, higher mortality, and longer hospital length of stay (LOS) than NH patients.

**Methods:**

This retrospective cohort study was conducted at a Level I Trauma Center and an American Burn Association-verified Regional Burn Center. We included adult patients admitted for pavement burns between January 1, 2015, and June 30, 2022. Patients in custody, pregnant patients, and those with missing data were excluded. Data were extracted from electronic medical records using a standardized tool by a blinded abstractor. The primary outcome was the percentage of total body surface area (TBSA) burned. Secondary outcomes included survival to hospital discharge and LOS. Data were analyzed using unpaired t-tests for continuous variables and Fisher’s exact test for proportions, with p< 0.05 considered statistically significant.

**Results:**

The study cohort included 305 patients, with 17.7% identified as PEH (n=54) compared to NH (n=251). The mean age of PEH was 51.2 years versus 54.6 years in NH, with no significant difference (95% CI = -1.69, 8.49; p=NS). PEH patients had a significantly lower mean BMI (24.8 vs. 28.6, diff ± SEM = 3.79 ± 1.12; 95% CI = 1.59, 5.99; p< 0.0001). There was no significant difference in TBSA (PEH = 5.81% vs. NH = 6.81%; 95% CI = -0.90, 0.96; p=NS), survival to discharge (PEH = 96.3% [95% CI = 86.7, 99.7] vs. NH = 89.1% [95% CI = 85.3, 93.5]; p=NS), or LOS (mean LOS: PEH = 22.7 days vs. NH = 19.1 days; 95% CI = -11.5, 4.4; p=NS).

**Conclusions:**

Our study found no significant differences in burn severity, mortality, or hospital LOS between PEH and NH patients with pavement burns. Despite this, homeless individuals remain at a higher risk for burn injuries due to increased exposure to extreme heat and lack of protective measures. These findings underscore the need for targeted public health interventions to prevent pavement burns, particularly in vulnerable populations.

**Applicability of Research to Practice:**

These results highlight the importance of developing targeted outreach and prevention programs as well as equitable emergency care protocols for vulnerable populations like persons experiencing homelessness who are at higher risk for pavement burns.

**Funding for the Study:**

N/A

